# AMPK Signaling Regulates the Age-Related Decline of Hippocampal Neurogenesis

**DOI:** 10.14336/AD.2019.0102

**Published:** 2019-10-01

**Authors:** Brian Z Wang, Jane J Yang, Hongxia Zhang, Charity A Smith, Kunlin Jin

**Affiliations:** ^1^Department of Pharmacology & Neuroscience, UNT Health Science Center, TX 76107, USA; ^2^School of Interdisciplinary Studies, University of Texas at Dallas, TX 75080, USA

**Keywords:** AMPK, metabolism, stem cell, aging, Compound C, AICAR

## Abstract

The global incidence of age-associated neurological diseases is expected to rise with increasingly greying societies. In the aged brain, there is a dramatic decrease in the number of stem cells, which is a main cause for the decrease in brain function. Intrinsic factors, such as cell metabolism, have been studied but its role in neurogenesis is still unknown. Therefore, this study sought to establish whether AMP-activated protein kinase (AMPK) signaling does indeed regulate hippocampal neurogenesis in the aged brain. We found that i) AMPKα2 was the predominant catalytic subunit in the subgranular and subventricular zones; ii) AMPK activation was at a significantly higher level in the aged *vs.* young hippocampus; iii) short term (7 days) treatment with selective AMPK signaling inhibitor Compound C (10 mg/kg/day, i.p.) significantly increased the numbers of newborn (BrdU^+^), Type 2 (MCM2^+^), and Type 3 (DCX^+^) neural stem cells, but not Type 1 (GFAP^+^/Sox2^+^) cells, in the aged hippocampus. Taken together, our results demonstrate that AMPK signaling plays a critical role in the age-related decline of hippocampal neurogenesis.

Aging is the progressive decline of physiological function and increased vulnerability to disease and death [1]. By the year 2050, 2 billion people i.e. one-fifth of the global population, will be over the age of 60 [2, 3]. With this increase in the proportion of elderly people, the incidence of age-associated neurological diseases such as Alzheimer’s disease and stroke are also expected to rise. Thus, there is an urgent need to find therapies to promote healthy brain aging.

The finding that neurogenesis still persists in adulthood guides the current stem cell and aging fields in targeting endogenous neurogenesis as a therapeutic for healthy brain aging [4, 5]. It is well established that progressive aging is associated with a dramatically increased susceptibility to neurodegenerative diseases [6]; a possible cause would be the dramatic decrease in neurogenesis with age [7]. The reasons for the age-related decline in neurogenesis can be due to cell intrinsic factors such as metabolism, which have been studied but its role in neurogenesis remains largely unexplored.

It has been reported that stem cells possess metabolically different characteristics from their differentiated progeny [8, 9]. Furthermore, proteomic analyses showed that almost half of the differentially expressed proteins identified in differentiated neurons *vs.* non-differentiated NSCs were proteins involved in metabolism, suggesting the need for a shift in cellular metabolism to accommodate the requirements for neurogenesis to occur [10, 11]. Indeed, cell metabolism and proliferation are closely interdependent processes [12]. Thus, an ideal candidate for the regulation of neurogenesis in the adult brain is AMP-activated protein kinase (AMPK) since it has been shown to single-handedly control a plethora of metabolic pathways [13, 14]. AMPK is a serine/threonine kinase and its structure and function in maintenance of energy equilibrium at the whole-body and cellular levels have been discussed [13, 15, 16]. However, its regulation of neurogenesis in the aged brain has not been studied, which is the aim of this study.

**Figure 1.**
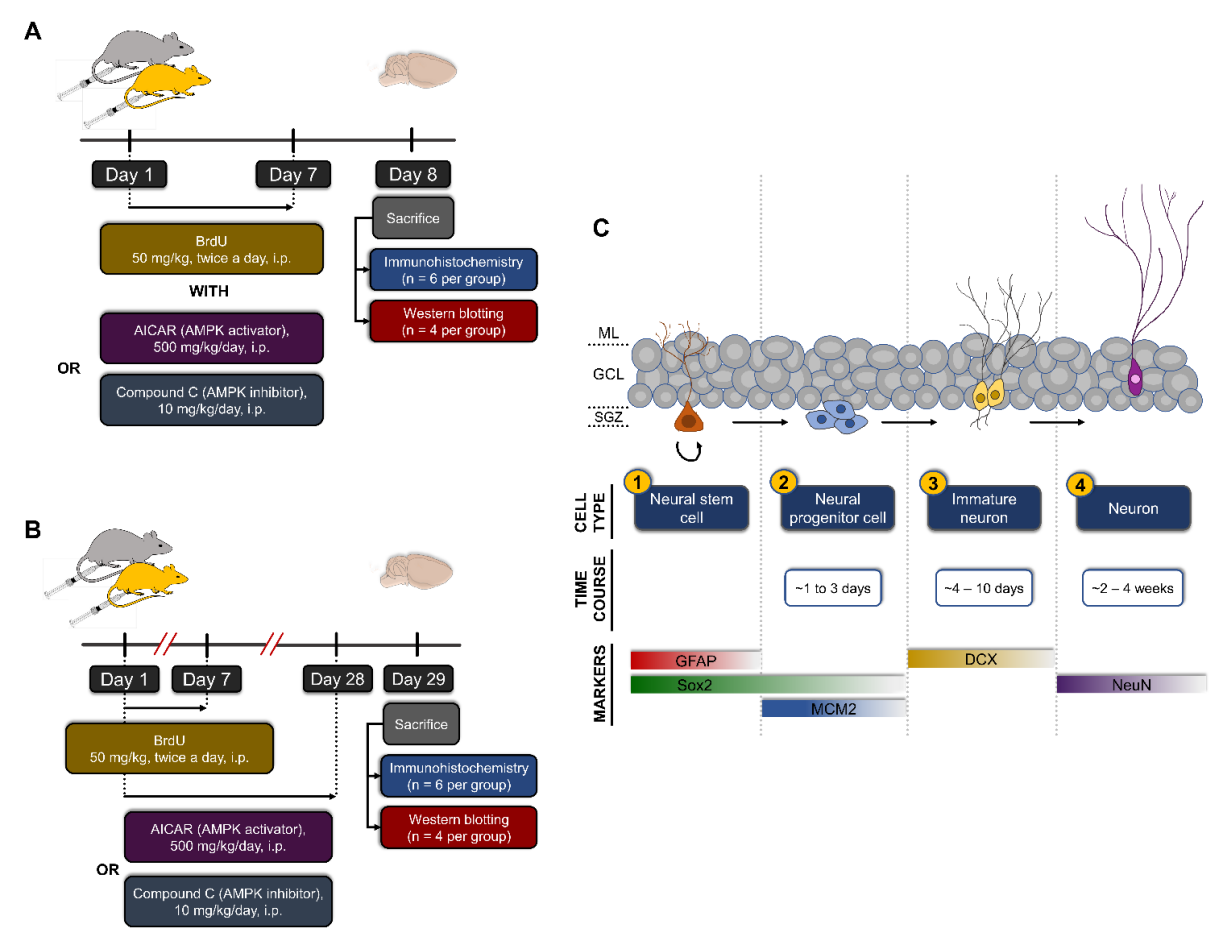
Experimental design. Young (2-3 months, orange mouse) and aged (19-20 months, grey mouse) male C57BL/6J mice were randomly divided into the groups outlined in the Materials and Methods section. The infographic here describes the design and timeline for studying the (A) short term and (B) long term effects of forced inhibition and activation of AMPK signaling on hippocampal neurogenesis. (C) Summary of the four stages during adult hippocampal neurogenesis: (1) quiescent radial glia-like (Type 1) cells in the subgranular zone (SGZ) are activated; (2) proliferation of non-radial progenitor (Type 2) cells; (3) generation of neuroblasts (Type 3); (4) maturation of neurons. Also shown are the time course for each stage and expression of stage-specific markers which were utilized for their identification in this study [17, 18]. DCX: doublecortin; GCL: granule cell layer; GFAP: glial fibrillary acidic protein; MCM2: minichromosome maintenance complex component 2; ML: molecular layer; NeuN: neuronal nuclei; SGZ: subgranular zone; Sox2: SRY (sex determining region Y)-box 2.

AMPK exists as a heterotrimer that can form different combinations of α, β, and γ subunits, which are encoded by distinct genes to produce two α subunits (α1 and α2) that mediate AMPK’s catalytic activity, two β (β1 and β2) and three γ (γ1, γ2, and γ3) subunits that regulate AMPK’s phosphorylation and activity [16, 19]. Various combinations of these subunit proteins can generate twelve heterotrimeric configurations of AMPK, whose structures are essential to cope with the diverse roles in regulating metabolic processes in response to various stimuli. AMPK is regulated by three main upstream kinases - liver kinase B1 (LKB1), calmodulin-dependent kinase kinase β (CaMKKβ), and the transforming growth factor beta-activated kinase 1 (TAK1). When energy (ATP) levels are low in the cell, AMPK is activated to restore energy to equilibrium by triggering energy-producing metabolic processes such as glycolysis and fatty acid oxidation, while simultaneously inhibiting energy-consuming metabolic pathways such as protein and fatty acid synthesis [13].

Since AMPK can control metabolic pathways to provide the building blocks for cell proliferation and have been shown to be implicated in aged tissues such as the brain [20], myocardium [21], and skeletal muscle [22, 23], we hypothesized that the inhibition of AMPK signaling in the aged brain will cause a concomitant increase in hippocampal neurogenesis. Here, we demonstrated that AMPK signaling activation was differentially expressed with age in the hippocampus and subventricular zone and uncovered a new role for the inhibition of AMPK signaling, namely its ability to increase hippocampal neurogenesis in the aged brain via short term pharmacological inhibition with Compound C, which suggests AMPK’s critical involvement in the regulation of downstream processes for the age-related decline in hippocampal neurogenesis.

## MATERIALS AND METHODS

### Ethics statement

All animal procedures were approved by the Institutional Animal Care and Use Committee at the University of North Texas Health Science Center (UNTHSC). The study was conducted according to the NIH Guide for the Care and Use of Laboratory Animals. Every effort was made to reduce the number of animals used as well as to minimize suffering to the animals.

### Chemicals and antibodies

5-Aminoimidazole-4-carboxamide ribonucleotide (AICAR, AMPK activator, Cat. # A611700) and 6-[4-(2-Piperidin-1-yl-ethoxy)-phenyl)]-3-pyridin-4-yl-pyrrazolo [1,5-a]-pyrimidine dihydrochloride (Compound C dihydrochloride, AMPK inhibitor, Cat. # CD0339) were obtained from Toronto Research Chemicals (ON, Canada) and Chemdea (NJ, USA), respectively. 5-bromo-2’-deoxyuridine (BrdU, Cat. # B5002) and paraformaldehyde (Cat. # P6148) were obtained from Millipore-Sigma (MO, USA).

Primary antibodies used are as follows: AMPKα1 and AMPKα2 (Cat. # ab3759, ab3760, respectively, both 1:200; Abcam, USA), AMPKβ1 and AMPKβ2 (Cat.# orb37351, orb381985, respectively, both 1:100; Biorbyt, UK), AMPKγ1, AMPKγ2, and AMPKγ3 (Cat. # orb247883, orb304519, orb37357, respectively, all 1:50; Biorbyt, UK), Phospho-AMPKα (Thr 172) (Cat.v# sc-33524, 1:200; Santa Cruz, USA), BrdU (Cat. # ab6326, 1:500; Abcam, USA), GFAP (Cat.v# 3670, 1:500; Cell Signaling, USA), Sox2 (Cat. # AF2018, 1:200; R&D Systems), MCM2 (Cat. # 3619, 1:200; Cell Signaling, USA), DCX (Cat. # sc-8066, 1:200; Santa Cruz, USA).

### Animals

A total of 126 young-adult (referred to as “young” from this point forth, 2-3 months; body weight 20-25 g, Charles River) and aged (19-20 months; body weight 35-42 g, National Institute of Aging) male C57BL/6J mice were randomly divided into the following schedules:

Schedule I - short term intraperitoneal drug administration for a duration of 7 days (Figure 1A): Young-vehicle-7d group (n = 10), Young-activator-7d group (n = 10), Young-inhibitor-7d group (n = 10); Aged-vehicle-7d group (n = 10), Aged-activator-7d group (n = 10), Aged-inhibitor-7d group (n = 10).

Schedule II - long term intraperitoneal drug administration for a duration of 28 days (Figure 1B): Young-vehicle-28d group (n = 10), Young-activator-28d group (n = 10), Young-inhibitor-28d group (n = 10); Aged-vehicle-28d group (n = 10), Aged-activator-28d group (n = 10), Aged-inhibitor-28d group (n = 10).

Schedule III - short term intracerebroventricular drug administration for a duration of 3 days: Young-vehicle group (n = 3), Young-activator group (n = 3).

For each group in Schedules I and II, n = 6 animals were allocated for immunohistochemical studies, while n = 4 animals were used for Western blotting. For each group in Schedule II, n = 4 mice were randomly selected to measure body weight and assess behavioral changes weekly (at 9 am). All animals were housed in the UNTHSC vivarium and maintained at 23 ± 1 °C on a 12-hr light/dark cycle starting at 0700 hours with *ad libitum* access to food and water.

### BrdU administration

To visualize newborn cells, mice used for immunohistochemical studies were intraperitoneally (i.p.) administered BrdU (50 mg/kg, twice a day) for seven days as described previously [24-26]. BrdU stock (10 mg/mL) was dissolved in 0.9% saline with its final pH adjusted to 7.4.

### Administration of AMPK signaling activator and inhibitor

0.9% saline was used as the vehicle for all experiments. Stock solutions of AICAR (100 mg/mL) and Compound C (2 mg/mL) were made by dissolving the drugs in 0.9% saline. The pH of AICAR and Compound C stock solutions were adjusted to 7.4 and 7.0, respectively, then aliquoted and stored in -20 °C until further use. Mice were administered AICAR (500 mg/kg/day) or Compound C (10 mg/kg/day) via the i.p. route for 7 or 28 consecutive days.

For intracerebroventricular administration, young male C57BL/6J mice were anesthetized and implanted with an Alzet® osmotic minipump (Cat. # 1003D, Durect Corporation, CA, USA). The cannula was placed into the right lateral ventricle: 1 mm lateral to the midline, 0.34 mm posterior to the bregma, and 3.5 mm deep into the pial surface. AICAR (4 mM) was dissolved in 0.9% saline and pH adjusted to 7.4 and 7.0, respectively. Each mouse was infused for 3 days with 1 μL/hour of either 0.9% saline (vehicle) or AICAR (activator). Mice were sacrificed on day 4, and their brain tissues were collected for Western blotting analyses.

**Figure 2.**
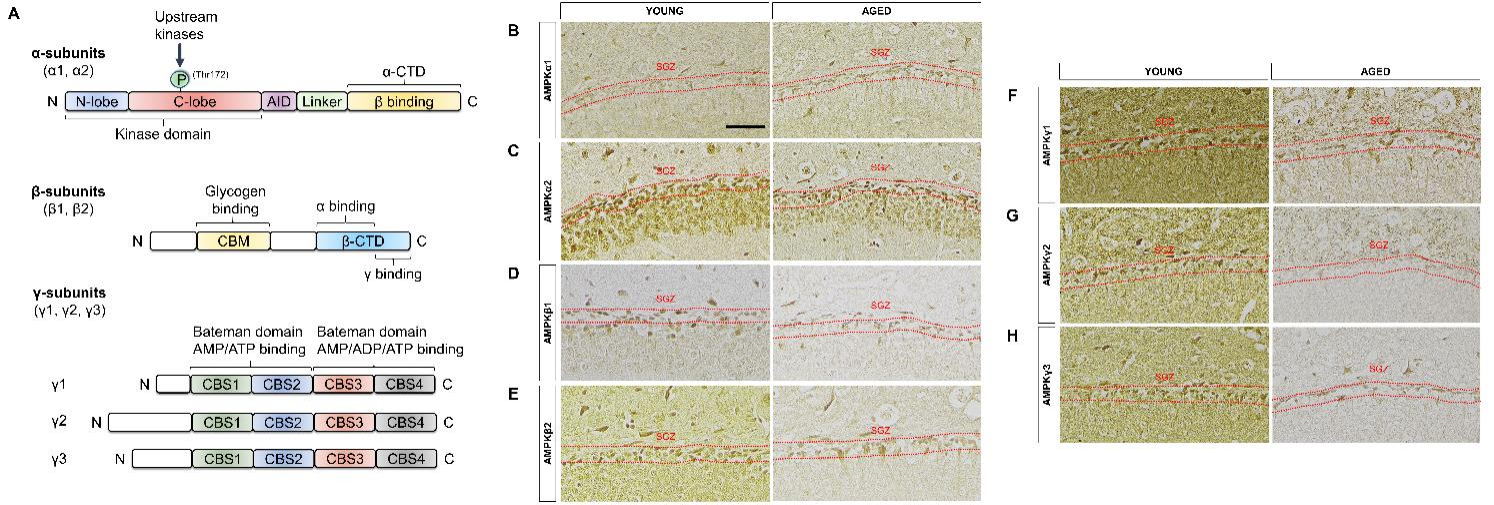
Expression patterns of AMPK subunit isoforms in the subgranular zone. (A) The AMPK complex is a heterotrimer made up of α, β, and γ subunits in a 1:1:1 ratio. The β-CTD of the β subunit forms the core of the complex, which binds to the N-terminus of the γ-subunit just before CBS1 and the α-CTD of the α-subunit. Representative images of coronal sections (5 μm) of young and aged mouse brains were immunostained to examine the expression pattern for all 7 AMPK subunit isoforms in the subgranular zone of the dentate gyrus (a 2-3 cell layer demarcated by the red dotted lines) where neurogenesis occurs. The representative images on the left show the expression patterns in the young brain while the right depict the patterns in the aged brain. All isoforms showed a lower expression level in the aged brain and were found in the cytoplasm with the exception of the α2 and β1 isoforms, which were localized to the nucleus. (B) AMPKα1, (C) AMPKα2, (D) AMPKβ1, (E) AMPKβ2, (F) AMPKγ1, (G) AMPKγ2, (H) AMPKγ3. Scale bar: 50 μm, n = 4 per group. AID: autoinhibitory domain; CBM: carbohydrate-binding module; CTD: carboxy-terminal domain; SGZ: subgranular zone.

### Immunohistochemistry

Mice were deeply anesthetized and sacrificed the day after their last injection. After perfusion with 0.9% saline and 4% PFA (pH adjusted to 7.4), their brains were collected, embedded in paraffin, and cut into 5 μm coronal sections.

Immunohistochemistry was performed as previously described [27] with modifications. In brief, sections were cleared in xylene, rehydrated through graded alcohols, and rinsed. These were then subjected to 0.3% Triton X-100 for 15 minutes, rinsed and incubated in citrate-based pH 6.0 antigen retrieval at 95-100 ºC for 10 minutes using the microwave method and allowed to cool to room temperature for an hour. To detect BrdU-labeled cells, sections were incubated in 2N HCl at 37 ºC for 30 minutes and rinsed with 0.1M boric acid (pH 8.5) for 10 minutes followed by PBS. To block endogenous peroxidase activity, sections were incubated in 3% H_2_O_2_ in ddH_2_O for 15 minutes after which they were blocked in blocking solution (3% horse serum, 1% bovine serum albumin in 1X PBS, pH 7.4) for 1 hour at room temperature and subsequently incubated with the appropriate primary antibodies at 4 ºC overnight (~16 hours). The following day, sections were washed with PBS and incubated with the appropriate horse biotinylated secondary antibodies (Cat. # BA-1100, BA-9500, both 1:200; Vector, CA, USA) for 1 hour at room temperature. After washing, the VECTASTAIN Elite ABC HRP solution was applied to the sections for 1 hour at room temperature. Finally, the HRP reaction was detected with DAB ImmPACT (Cat. # SK-4105, Vector, CA, USA) until the desired brown staining intensity is achieved. The reaction was stopped by rinsing the sections under running tap water for 5 minutes. Sections were then dehydrated, cleared in xylene, cover-slipped with permanent mounting medium (Vector, CA, USA), and air-dried overnight. Sections were examined and photographed with a Nikon Ti-E microscope and Nikon DS-Fi1 color camera (Nikon, NY, USA). Controls included omitting the primary antibodies.

### Multi-label immunostaining

Multi-label immunostaining was performed as previously described [27] with modifications. Briefly, sections were blocked in blocking solution (3% donkey serum, 1% bovine serum albumin (BSA) in 1X PBS, pH 7.4) for 1 hour at room temperature and incubated with the appropriate primary antibodies at 4 ºC overnight. Sections were then washed with PBS, incubated with the appropriate donkey (Cat. # A-11055, R37118, R37115, all 1:500) and rabbit (Cat. # A-31573, 1:50) secondary antibodies (Life Technologies, USA) for 1 hour at room temperature, rinsed, and cover-slipped with Prolong Gold Antifade with DAPI, according to the manufacturer’s instructions (Cat. # P36931, Life Technologies, USA). The sections were cured overnight in the dark. Controls included omitting the primary antibodies. Fluorescence signals were detected using a Nikon Ti-E microscope and Nikon DS-Qi MC camera (Nikon, NY, USA), and images were acquired using the NIS-Elements Basic Research software (Nikon, NY, USA).

### Cell counting

An observer blinded to the experimental conditions counted BrdU^+^, MCM2^+^, DCX^+^, and double-labeled (GFAP^+^/Sox2^+^) cells in the dentate gyrus of the hippocampus from four to six coronal sections per animal (n = 5 to 6 animals per group) spaced 100 µm apart beginning at bregma -1.64 mm. An average cell count was obtained by totaling the number of cells from each coronal section then dividing by the total number of coronal sections. The results are expressed as a percentage of the control.

### Western Blot analysis and densitometry

Mice were deeply anesthetized and sacrificed by cervical dislocation the day after their last injection. The subventricular zone (SVZ) and hippocampus were dissected on ice from young and aged mice as previously described [28], using a Zeiss V8 dissecting microscope. The brains were removed, placed in a coronal brain matrix, and cut into 1 mm thick coronal sections throughout the area of the SVZ (from bregma -0.30 to -1.2 mm), while the entire hippocampus was dissected by removing the surrounding cortex that encapsulates it. The dissected SVZ and hippocampi were flash frozen immediately in liquid nitrogen and stored at -80 °C for further analysis. Each dissection was timed from the start of cervical dislocation and performed within 8-10 minutes. The timing was crucial because AMPK signaling activation reaches its peak at 15 minutes [29].

Tissues were lysed through sonication using lysis buffer (13.5 mM NaCl, 2.7 mM KCl, 4.3 mM NaPO_4_, 1.4 mM KPO_4_, 0.5% NP-40, 0.5% sodium deoxycholate, pH 7.4) containing 1X Halt^TM^ protease and phosphatase inhibitor cocktail (Cat. # 78444, Thermo Fisher Scientific, USA). Protein concentration was determined using the Pierce 660 nm protein assay (Cat. # 22660, Thermo Fisher Scientific, USA) with BSA as a standard. Protein samples (30 µg) were boiled at 95-100 °C for 10 minutes, resolved on 10% SDS-PAGE gels, transferred onto polyvinylidene difluoride (PVDF) membranes, blocked with 5% non-fat dry milk in TBST (containing 0.1% Tween-20), and incubated overnight (~14 hours) with gentle shaking at 4 °C with either of the following primary antibodies (Cell Signaling Technology, MA, USA): (1) rabbit monoclonal anti-phospho-AMPKα (Thr 172) (Cat. # 2535, 1:1000), (2) rabbit monoclonal anti-AMPKα (Cat. # 5831, 1:1000), (3) rabbit monoclonal anti-pan-Actin (Cat. # 8456, 1:10,000).

Membranes were then washed with TBST, incubated at room temperature for two hours with horseradish peroxidase conjugated anti-rabbit secondary antibody (Cell Signaling Technology, 1:5000), and washed with TBST. Peroxidase activity was visualized by chemiluminescence with SuperSignal West Femto Maximum Sensitivity Substrate (Cat. # 34095, Thermo Fisher Scientific, USA) using Bio-Rad’s Chemidoc MP imaging system. Antibodies were then removed by incubating with a homemade stripping buffer (0.2 mM Glycine, 0.1% SDS, ddH_2_O, adjusted to pH 2.0 with HCl) with gentle rocking at room temperature for 30 minutes. Following washing with TBST, the membranes were blocked and reprobed with Actin. Densitometry measurements were obtained using the Image Lab v. 6.0 software by Bio-Rad. Bands were automatically selected by the software and default background subtraction of disk size 10.0 mm was applied to all bands. The “Adjusted Total Band Volumes” were then normalized to the densities of the housekeeping gene, Actin, of the same lane and blot to obtain relative expression.

### Open field test

Spontaneous locomotor activity (LMA) was measured weekly for a period of 28 days using the ANY-Maze (v. 5.3, Stoelting Co., IL, USA) software. During a 5-minute test period, the mouse's movement was recorded by a camera linked to the software to yield variables that described horizontal components of spontaneous activity such as distance traveled in meters.

### Data analysis

Sample size calculation using a power of 0.8 was determined with G*Power (v. 3.1, Universität Düsseldorf, Germany) [30]. Statistical analyses were performed using GraphPad Prism 7 (GraphPad Software, CA, USA). Absolute cell counts were subjected to a two-way analysis of variance (ANOVA) followed by Fisher’s Least Significant Difference (LSD) post hoc test with Treatment (Vehicle, Inhibitor or Activator) and Treatment duration (7 or 28 days) as between-groups factors. Body weight and spontaneous LMA were subjected to two-way repeated measures ANOVA with Treatment (Vehicle, Inhibitor or Activator) and Treatment duration (7 or 28 days) as between-groups factors. Densitometry measurements between two groups of mice (Young and Aged) were subjected to the Student's *t*-test. All data are expressed as mean ± standard error (SEM). A *p* value of less than 0.05 was regarded as statistically significant.

## RESULTS & DISCUSSION

### Age-related changes in AMPK subunit isoform expression and signaling

**Figure 3.**
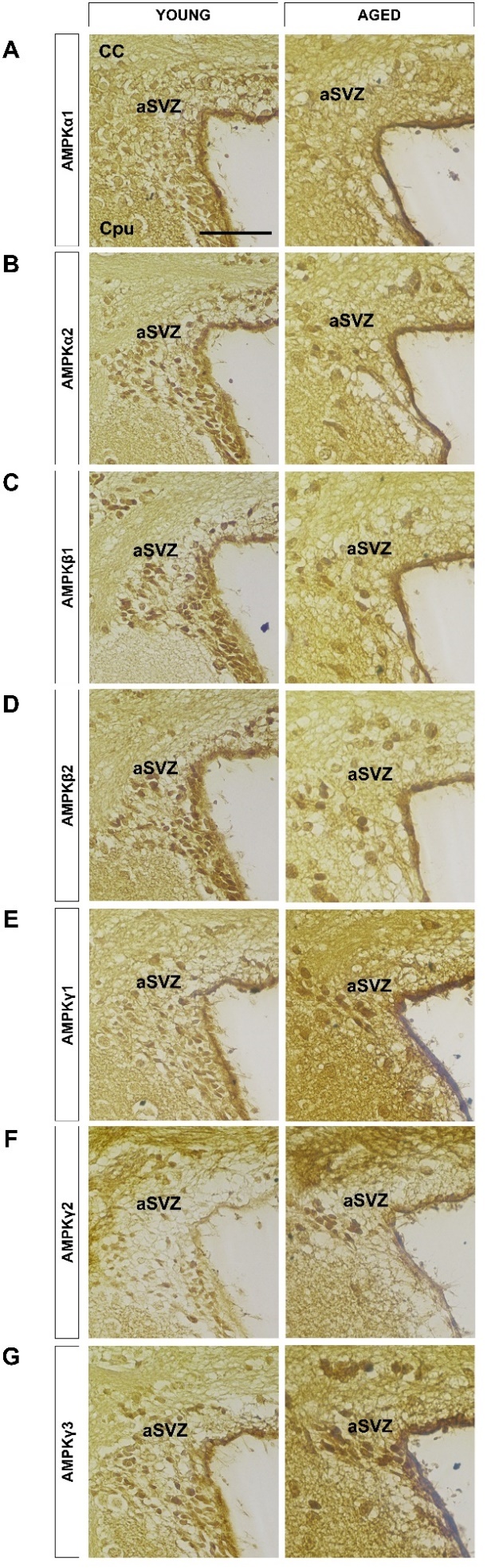
Expression patterns of AMPK subunit isoforms in the subventricular zone. Representative images of coronal sections (5 μm) of young and aged mouse brains were immunostained to examine the expression pattern for all 7 AMPK subunit isoforms in the subventricular zone (SVZ) of the lateral ventricles where neurogenesis occurs. The anterior SVZ (aSVZ) shown here has the highest concentration of neural stem cells. The representative figures on the left show the expression patterns in the young brain while the right depict the patterns in the aged brain. All isoforms were localized to the nucleus with the exception of α1, which was found in the cytoplasm. All α and β isoform levels decreased with age with the exception of all three γ isoforms that experienced increased levels with age. (A) AMPKα1, (B) AMPKα2, (C) AMPKβ1, (D) AMPKβ2, (E) AMPKγ1, (F) AMPKγ2, (G) AMPKγ3. Scale bar: 50 μm, n = 4 per group. aSVZ: anterior subventricular zone; CC: corpus callosum; CPu: caudate putamen (striatum).

The heterotrimeric complex of AMPK is made up of different subunit isoforms in the ratio of 1:1:1 (α:β:γ), which can result in the formation of at least twelve different AMPK complexes. These subunits could be differentially expressed in a tissue-specific manner with distinct subcellular localization (nuclear *vs.* cytoplasmic, or both) in various tissues, which might be important in regulating specific responses at the cellular and whole-body level. To date, there is only one other study that performed an extensive interrogation of AMPK isoform expression pattern and localization in the mouse central nervous system [31], while others merely focused on a specific isoform in different parts of the brain [19, 32-35]. Thus, information regarding their expression patterns in the two main adult neurogenic niches, the subgranular zone (SGZ, Figure 2) and SVZ (Figure 3), of young and aged mice were deficient. Our study is the first to demonstrate and compare the effects of age on the expression pattern of all seven AMPK subunit isoforms in the SGZ and SVZ.

**Figure 4.**
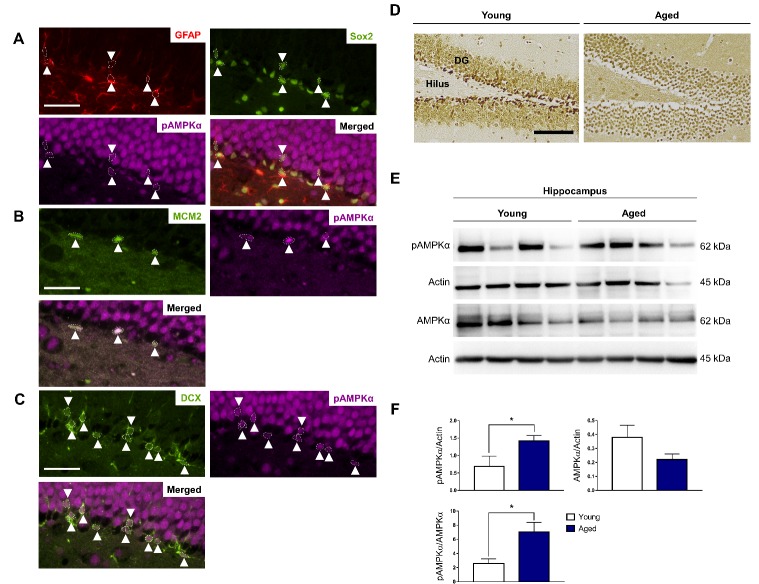
AMPK signaling activation is increased in the hippocampus with age. Representative images of coronal sections (5 μm) of young mouse brains were subjected to double- or triple-labeling to demonstrate the colocalization of AMPK signaling activation (pAMPKα) with individual stem cell types (indicated by white arrows) found in the subgranular zone: (A) quiescent Type 1 stem cells (GFAP^+^/Sox2^+^), (B) actively proliferating Type 2 progenitor cells (MCM2^+^), (C) differentiated immature neuroblasts (DCX^+^). Scale bars in A-C: 50 μm, n = 4 per group. (D) Representative images demonstrating AMPK signaling activation levels in the young and aged dentate gyrus. Scale bar: 100 μm, n = 4 per group. (E) Protein lysates (30 μg) obtained from young and aged hippocampi were resolved on 10% SDS-PAGE gels, transferred onto PVDF membranes and probed with the appropriate primary and secondary antibodies. Representative Western blots show pAMPKα and total AMPKα protein levels. Actin was used as the loading control. (F) Statistical analysis of relative protein levels of pAMPKα in the young and aged hippocampus showing a significant increase in AMPK signaling activation in the aged hippocampus. Data shown as mean ± standard error (SEM), n = 4 per group, *p < 0.05, Student’s *t*-test. DCX: doublecortin; DG: dentate gyrus; GFAP: glial fibrillary acidic protein; MCM2: minichromosome maintenance complex component 2; Sox2: SRY (sex determining region Y)-box 2.

Overall, the expression levels of all isoforms in the SGZ and SVZ were higher in the young *vs.* aged mice with the exception of i) pAMPKα in the aged SGZ and SVZ, and ii) AMPKγ isoforms in the aged SVZ. The activation of AMPKα is critical in mediating downstream signaling cascades, which is dependent on the isoform’s location. Once activated, in the cytoplasm, it triggers metabolic processes such as fatty acid oxidation and glycolysis while simultaneously inhibiting fatty acid synthesis and gluconeogenesis. If in the nucleus, it is involved in regulating gene expression [36, 37]. The AMPKα isoforms showed the expected localization pattern demonstrated by Turnley et al. [31] and others [38-40]. In the SGZ and SVZ, AMPKα1 was mainly localized to the cytoplasm (Figures 2B & 3A, respectively) while AMPKα2 was preferentially localized to the nucleus (Figures 2C & 3B, respectively), suggesting that during neurogenesis, α1 controls cytoplasmic metabolic processes while α2 governs AMPK-mediated transcriptional events.

**Figure 5.**
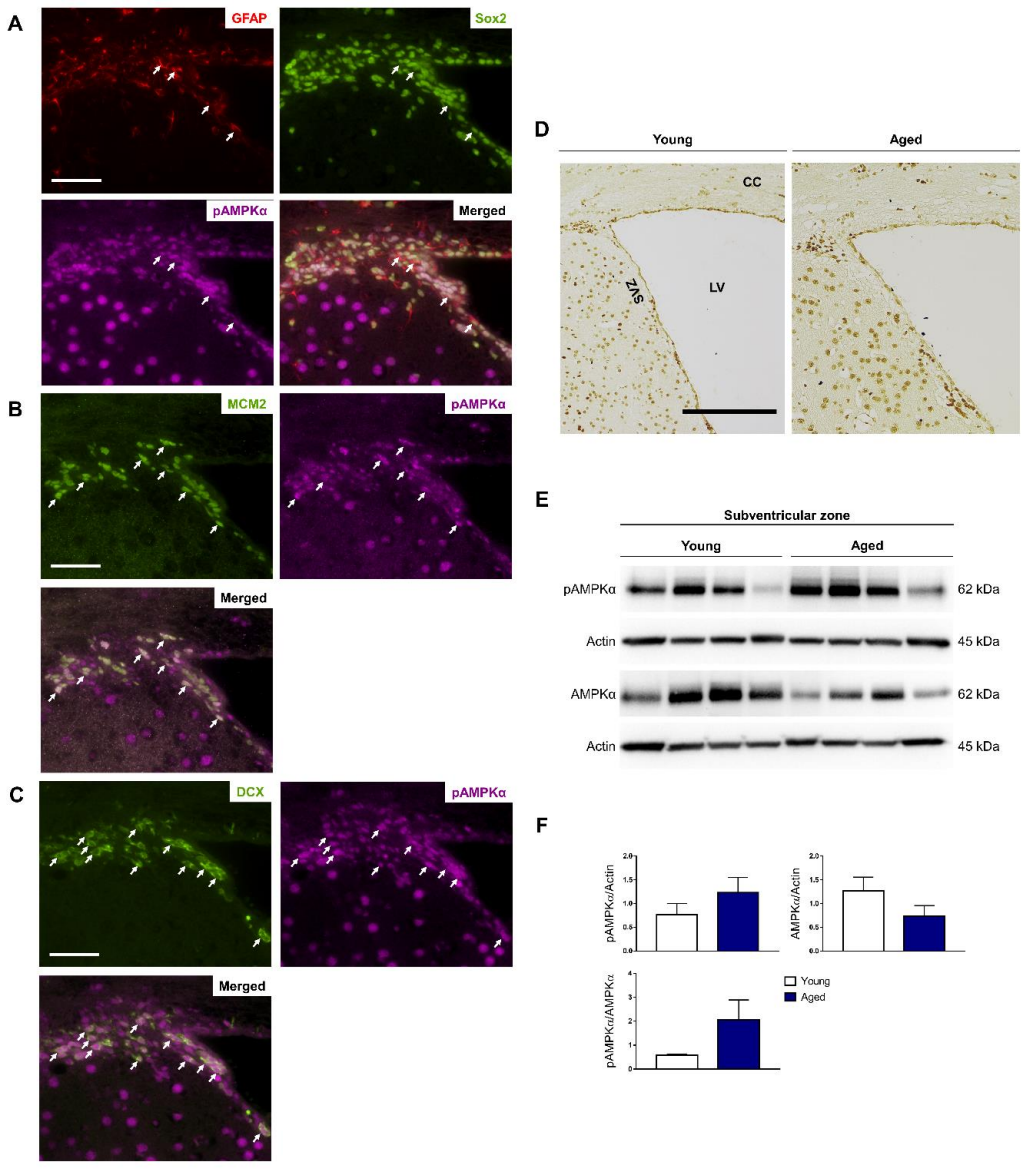
AMPK signaling activation is increased in the subventricular zone with age. Representative images of coronal sections (5 μm) of young mouse brains subjected to demonstrate the colocalization of AMPK signaling activation (pAMPKα) with individual stem cell types (indicated by white arrows) found in the SVZ: (A) quiescent Type 1 stem cells (GFAP^+^/Sox2^+^), (B) actively proliferating Type 2 progenitor cells (MCM2^+^), (C) differentiated immature neuroblasts (DCX^+^). Scale bars in A-C: 50 μm, n = 4 per group. (D) Representative images demonstrating AMPK signaling activation levels in the young and aged SVZ. Scale bar: 100 μm, n = 4 per group. (E) Protein lysates (30 μg) obtained from microdissected young and aged SVZ were resolved on 10% SDS-PAGE gels, transferred onto PVDF membranes and probed with the appropriate primary and secondary antibodies. Representative Western blots show pAMPKα and total AMPKα protein levels. Actin was used as the loading control. (F) Statistical analysis of relative protein levels of pAMPKα in the young and aged SVZ showing an increase in AMPK signaling activation with age. Data shown as mean ± standard error (SEM), n = 4 per group, Student’s *t*-test. CC: corpus callosum; DCX: doublecortin; GFAP: glial fibrillary acidic protein; LV: lateral ventricle, MCM2: minichromosome maintenance complex component 2; Sox2: SRY (sex determining region Y)-box 2; SVZ: subventricular zone.

Our finding regarding the abundance of α2 expression in the SGZ and SVZ corroborates with the idea that it is the predominant catalytic isoform in the brain [31]. The importance of the brain catalytic α2 isoform and by extension AMPK activation, have been implicated in age-related neurological disorders: i) pharmacological inhibition of AMPK activity by Compound C [41] and deletion of α2 in the brain provided neuroprotection after ischemic stroke [42]; ii) AMPK was hyper-activated in Alzheimer’s disease (AD) human brains [43] and AMPKα2^-/-^ mice had a reduction of endogenous tau phosphorylation [44] and inhibited Aβ-induced LTP failure [45]. pAMPKα was localized to the nucleus in all stem cell types found in the SGZ (Figure 4A-C) and SVZ (Figure 5A-C), suggesting its involvement in neurogenesis. The expression levels for pAMPKα was found to be consistently higher in the aged *vs.* young SGZ and SVZ (Figure 4D-F and Figure 5D-F, respectively). A study conducted using human subjects (n = 120) found that there was an age-related increase in resting metabolic rate in the brain [46]. Thus, with increased energy consumption in the aged brain, AMPK signaling is activated to a greater extent, which corroborates with our data.

Besides binding the α and γ subunits through its C-terminal domain to allow the formation of a stable heterotrimer (Figure 2A), another important role of the regulatory AMPKβ subunit is its ability to regulate AMPK substrate selection by directing the α subunit to its cellular compartment [47, 48]. It was surprising that both β isoforms in the SGZ (Figure 2D-E) and SVZ (Figure 3C-D) were found in the nucleus, which suggests that the α2 isoform is able to associate with either of the β isoforms regardless of cell compartment. This idea of subunit selection is supported by Chen et al. where they performed immunoprecipitation and found that α2 in the extensor digitorum longus (EDL) muscle could associate with both β1 and β2 while α2 in the soleus muscle only associated with the β1 isoform. Furthermore, β2 was found to associate with α2 only and not α1. However, the factors promoting the association of α2 with β1 or β2 isoforms are still unknown [49].

The regulatory AMPKγ subunit acts as an allosteric activator of AMPK. It contains two Bateman domains (Figure 2A) that allow the binding of AMP and ADP to result in the allosteric activation of AMPK. The γ isoforms showed higher expression levels in the aged *vs.* young SVZ. This could have been better substantiated with an additional method such as Western blotting, which is useful for semi-quantitatively measuring protein expression levels. Strikingly, we observed a region-specific difference in the localization of γ isoforms in the SGZ and SVZ. All γ isoforms in the SGZ were found in the cytoplasm (Figure 2G-H) while those in the SVZ were localized to the nucleus (Figure 3E-G). Furthermore, the expression levels of γ isoforms in the aged SVZ were higher compared with the aged SGZ. The localization of all γ isoforms in one cellular compartment has not been reported.

Concerning the β and γ subunits, the question remains as to whether AMPK subunit isoforms could be present in the same subcellular location, form a functional complex in a different compartment, and still carry out their intended functions. One possibility is that the individual isoform is using a shuttling system to transport it to its required location and bring it back to its original subcellular compartment. It can be thought as energy inefficient yet it has been shown that tRNAs in yeast can be shuttled into the cytosol and back to the nucleus [50]. Another study supports this nucleocytoplasmic transport reversibility and went further to demonstrate that shuttling is a fundamental feature when the accumulation of molecules in one cellular compartment was observed [51]. One other probable reason relates to changes in the cell being dynamic and fast-paced. Since AMPK is known to be the master regulator of cellular metabolic processes, it needs to be quick and sensitive to respond to energy changes in the cell. Therefore, it is likely that the concept of subunit isoform switching, which is well-known in the field of receptor pharmacology, can be applied in this case especially when the isoforms are aggregated or localized to the same cellular compartment. Analogous to how a sport such as basketball can have an unlimited number of player substitutions to cope with changing situations on the court, AMPK, when faced with a certain stressor such as low energy levels in the cell, through a yet unknown mechanism of isoform selection, can then choose which isoforms to recruit and switch out to ensure its maximum effectiveness of dealing with that stressor e.g., as discussed earlier, the α2 isoform was able to associate with either of the β isoform in the EDL muscle but only associates with the β1 isoform in the soleus muscle [49]. It is also plausible to marry the two notions above for explaining the conundrum. AMPK isoforms can rely on reversible nucleocytoplasmic transport and form the most appropriate heterotrimeric AMPK complex for activating various downstream metabolic processes. Therefore, we hypothesize that the formation of the AMPK complex is dynamic and not static, an idea that has been recently shown to be possible for other protein complexes [52, 53].

**Figure 6.**
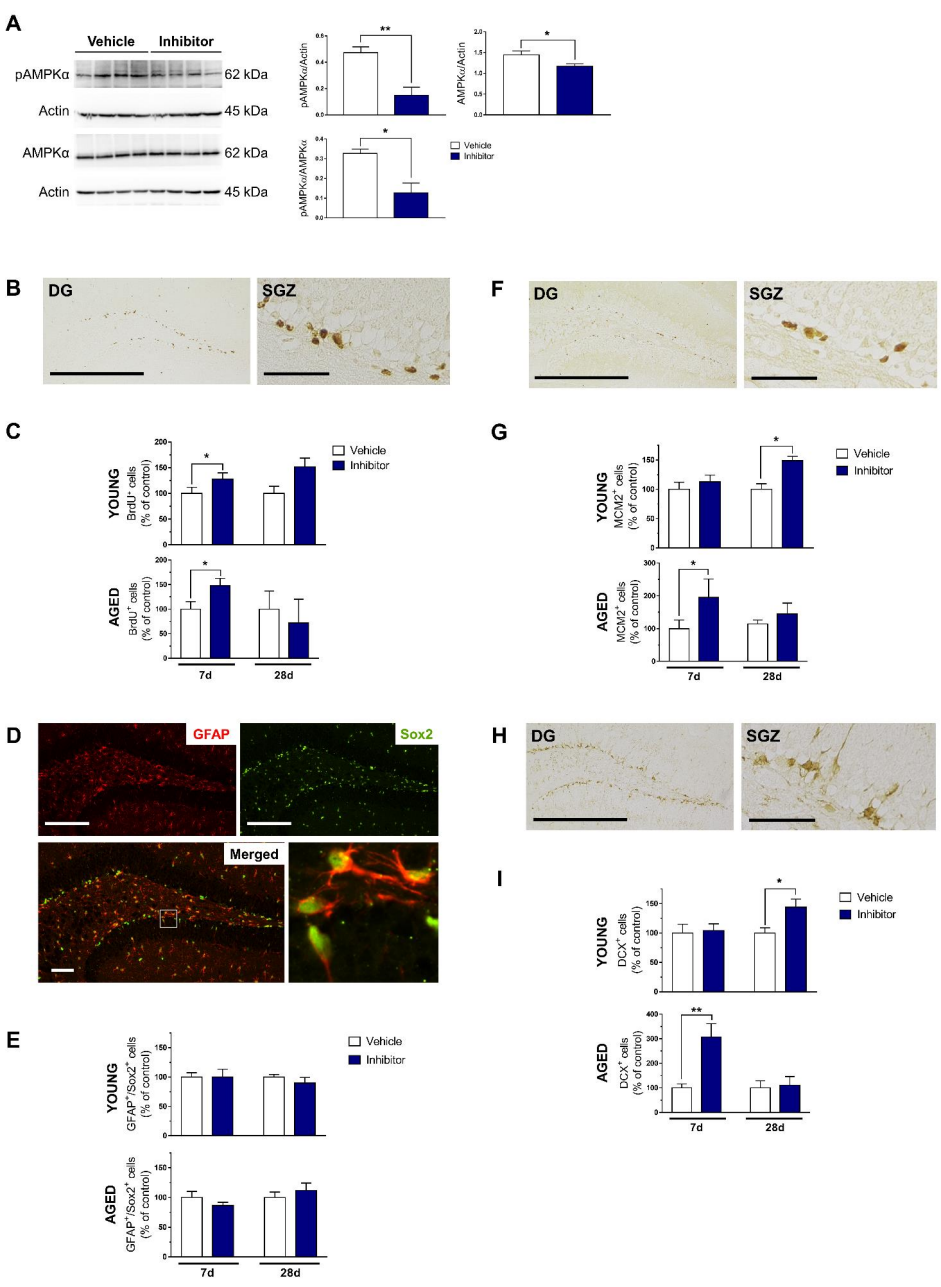
Pharmacological inhibition of AMPK signaling with Compound C increases hippocampal neurogenesis. Young and aged mice were subjected to intraperitoneal administration of vehicle (0.9% saline) or specific AMPK inhibitor (Compound C, 10 mg/kg/day) for 7 and 28 days. (A) Protein lysates (30 μg) from the young vehicle and inhibitor groups were resolved on 10% SDS-PAGE gels, transferred onto PVDF membranes and probed with the appropriate primary and secondary antibodies. Representative Western blots show pAMPKα and total AMPKα protein levels. Actin was used as the loading control. Statistical analysis of relative protein levels of pAMPKα in the young hippocampus indicating that Compound C mediated neurogenesis was AMPK-dependent. Data shown as mean ± standard error (SEM), n = 4 per group, *p < 0.05, **p < 0.01, Student’s *t*-test. Representative immunostaining for (B) newborn BrdU^+^ neural stem cells (NSCs), (D) GFAP^+^/Sox2^+^ Type 1 NSCs, (F) MCM2^+^ Type 2 NSCs, and (H) DCX^+^ Type 3 NSCs in the DG and SGZ of young mice. Scale bars in left panels of (B), (F), (H): 500 µm, Scale bars in right panels of (B), (F), (H): 50 µm, Scale bars in (D): top panel, 200 µm; bottom panel, 50 µm. Quantification of (C) newborn, (E) Type 1, (G) Type 2, and (I) Type 3 NSCs in the young and aged SGZ after short- and long-term treatment with Compound C. Data shown as mean ± standard error (SEM), n = 5-6 mice/group/time point, 4-6 sections per animal, *p < 0.05, **p < 0.01, Two-way ANOVA (between-groups factors: treatment, treatment duration) followed by Fisher’s LSD post hoc test. DCX: doublecortin; DG: dentate gyrus; GFAP: glial fibrillary acidic protein; MCM2: minichromosome maintenance complex component 2; SGZ: subgranular zone; Sox2: SRY (sex determining region Y)-box 2.

### Effects of forced inhibition of AMPK signaling on hippocampal neurogenesis

To examine the effects of the loss of function of AMPK activation on the age-related decline in hippocampal neurogenesis, young and aged mice were subjected to short term (7 days) and long term (28 days) i.p. administration of the specific AMPK inhibitor, Compound C. It is a well-known and widely used inhibitor of AMPK signaling that acts as an ATP-competitive inhibitor [54] by binding reversibly to the AMPKα subunit kinase domain [55]. A dose of 10 mg/kg/day was sufficient to induce a significant inhibition in AMPK signaling (*p* < 0.05, *vs.* vehicle) (Figure 6A). We then evaluated the number of newborn cells (BrdU^+^) as well as Type 1 (GFAP/Sox2^+^), Type 2 (MCM2^+^), and Type 3 (DCX^+^) cells after 7 and 28 days of i.p. treatment. With respect to newborn cells, there were significant increases in the inhibitor-treated young (*p* < 0.01, *vs.* vehicle) and aged (*p* < 0.05, *vs.* vehicle) groups after short term administration (Figure 6C). No significant changes were seen for Type 1 cells (Figure 6E). The inhibitor-treated young group had about 50% increase in Type 2 cells after long term treatment (*p* < 0.05, *vs.* vehicle) while the inhibitor-treated aged group had approximately 100% increase after short term treatment (*p* < 0.05, *vs.* vehicle) (Figure 6G). Likewise, inhibitor-treated young mice experienced about 50% increase in Type 3 cells after long term treatment (*p* < 0.05, *vs.* vehicle) while aged mice had an approximate 200% increase after short term treatment only (*p* < 0.01, *vs.* vehicle) (Figure 6I). Taken together, the increase in neurogenesis mediated by Compound C was AMPK-dependent. Furthermore, it seems at this point that inhibition of AMPK signaling is very likely to be involved in augmenting the proliferative events of the neurogenic process. Indeed, this is congruent with the idea that when AMPK signaling is inhibited (decrease in AMPK signaling activation), downstream metabolic pathways such as glycolysis and fatty acid oxidation are downregulated while protein and fatty acid synthesis are upregulated, which allow for cell proliferation. The short term and long term effects of Compound C on cell proliferation have not been studied *in vivo*. It has been suggested that the age-related decrease in neurogenesis may be a consequence of several processes that control NSC dynamics such as quiescence, terminal differentiation, increase in cell cycle length, senescence or death [56]. One study reported that decreased neurogenesis in the aged mouse was due to proliferating NSCs becoming quiescent via the Wnt signaling pathway and they found that this process was reversible [57]. However, this was not true in our case as Type 1 cells, which are quiescent, did not increase with short term and long term Compound C treatment. Moreover, it could not have been due to the prolongation of the cell cycle length since we did not witness an increase in cell proliferation in Types 2 and 3 cells with long term Compound C treatment. Thus, we speculate that with already low numbers and very limited divisions remaining in aged hippocampal NSCs, inhibition of AMPK signaling increased cell proliferation in the short term only possibly due to the exhaustion of the stem cell pool.

### Effects of forced activation of AMPK signaling on hippocampal neurogenesis

To study the effects of the gain of function of AMPK activation on the age-related decline in hippocampal neurogenesis, young and aged mice were subjected to short and long term administration of the AMPK activator, AICAR, which is an analog of adenosine that is taken up by cells through adenosine transporters then phosphorylated by adenosine kinase, giving rise to the AMP-mimetic, AICAR monophosphate (ZMP) [58].

**Figure 7.**
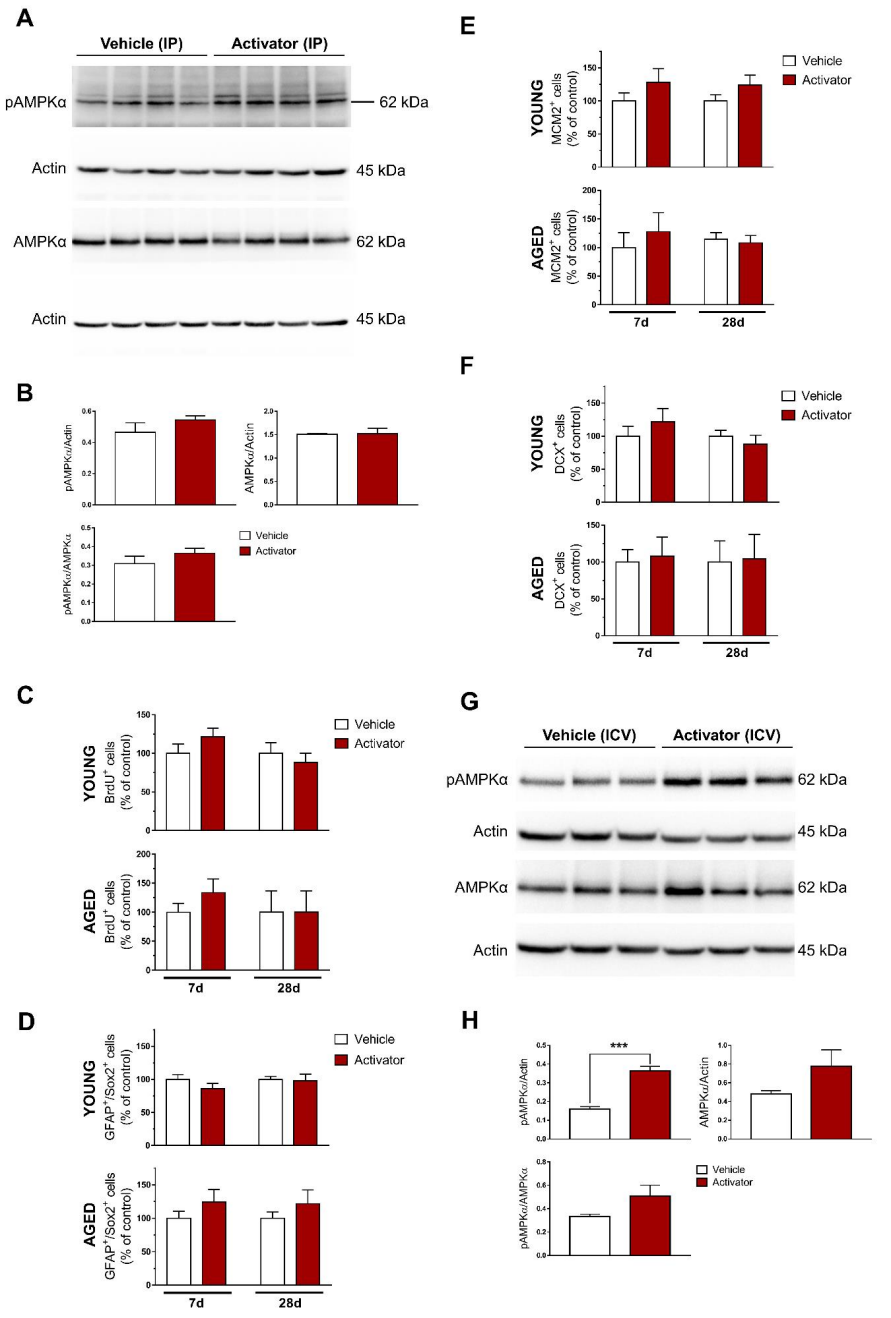
Pharmacological activation of AMPK signaling with AICAR does not impact hippocampal neurogenesis in the male mouse. Young and aged mice were subjected to intraperitoneal administration of vehicle (0.9% saline) or specific AMPK activator (AICAR, 500 mg/kg/day) for 7 and 28 days. (A) Protein lysates (30 μg) from the young vehicle and activator groups were resolved on 10% SDS-PAGE gels, transferred onto PVDF membranes and probed with the appropriate primary and secondary antibodies. Representative Western blots show pAMPKα and total AMPKα protein levels. Actin was used as the loading control. (B) Statistical analysis of relative protein levels of pAMPKα in the young hippocampus revealed that AICAR failed to activate AMPK signaling. Data shown as mean ± standard error (SEM), n = 4 per group, Student’s *t*-test. Quantification of (C) newborn, (D) Type 1, (E) Type 2, and (F) Type 3 NSCs in the young and aged SGZ after short- and long-term treatment with AICAR. Data shown as mean ± standard error (SEM), n = 5-6 mice/group/time point, 4-6 sections per animal, Two-way ANOVA (between-groups factors: treatment, treatment duration) was performed but we found no significant interactions. (G-H) To rule out the possibility of obtaining a bad lot of the drug, we administered AICAR (4 mM) centrally for 3 days using a minipump with flow rate of 1 μL/hr and found that AICAR could indeed activate AMPK signaling, raising the possibility that AICAR was not able to cross the blood-brain-barrier in the male mouse. Data shown as mean ± standard error (SEM), n = 3 per group, ***p < 0.001, Student’s *t*-test. DCX: doublecortin; GFAP: glial fibrillary acidic protein; ICV: intracerebroventricular; IP: intraperitoneal; MCM2: minichromosome maintenance complex component 2; Sox2: SRY (sex determining region Y)-box 2.

Its mechanism of action is similar to that of AMP where it allosterically activates AMPK by binding to the cystathione-β-synthase (CBS) domain 3 on the AMPKγ subunit (Figure 2A). AICAR acts as a direct activator of AMPK in that it does not alter the ADP:ATP ratio or oxygen uptake like many other AMPK activators do by inhibiting mitochondrial function [59]. Therefore, AICAR was used as our AMPK activator of choice. We expected AMPK signaling to increase in the young hippocampus and consequently observe a decrease in neurogenesis. Further, we expected no change to cell proliferation in the aged hippocampus since an increasing body of evidence have already shown that AMPK is not as sensitive to changes in the cell and could not effectively respond to the stimuli with advancing age [20, 22, 60]. Surprisingly, we observed i) an absence of increased AMPK signaling compared with vehicle (Figure 7A) and ii) no decrease in neurogenesis in the young hippocampus (Figure 7B-F).

To rule out the possibility of a bad lot of the drug, we infused AICAR (4 mM) for 3 days using a minipump that delivers the drug directly into the brain and found a robust increase in AMPK signaling (*p* < 0.0001, *vs.* vehicle) (Figure 7G-H). Interestingly, two separate reports from the same group using the same dose (500 mg/kg/day) and route of administration (via i.p.), demonstrated that AICAR had an effect on neurogenesis in young and aged female mice [61, 62]. The authors cited a report showing that only less than 1% of AICAR could cross the blood-brain barrier [63], which could have contributed to the absence of increased AMPK signaling activation in our male mouse model. Possible reasons for this disparity suggest that i) the AMPK complex in the female mouse is more responsive to the less than 1% of AICAR that crossed the blood-brain-barrier compared with the male mouse of the same strain and age; ii) there may be a sex-specific difference in the composition of the blood brain barrier. Indeed, it was reported that the sphingosine 1-phosphate receptor 2 (S1PR2) found in the central nervous system vasculature was exclusive to female SJL EAE mice and female patients with multiple sclerosis. S1PR2 destabilizes adherens junctions in endothelial cells of the blood-brain-barrier and was demonstrated to be higher in females rather than males [64]. Another study showed that S1PR2 is exclusively upregulated in microvessels in stroke to induce cerebrovascular permeability [65]. Taken together, females seem to have a more permeable blood-brain-barrier compared with males, which could have allowed for a considerable amount of AICAR to cross into the brain and effect significant changes.

### Effects of long term administration of AMPK signaling activator and inhibitor on vital signs

**Figure 8.**
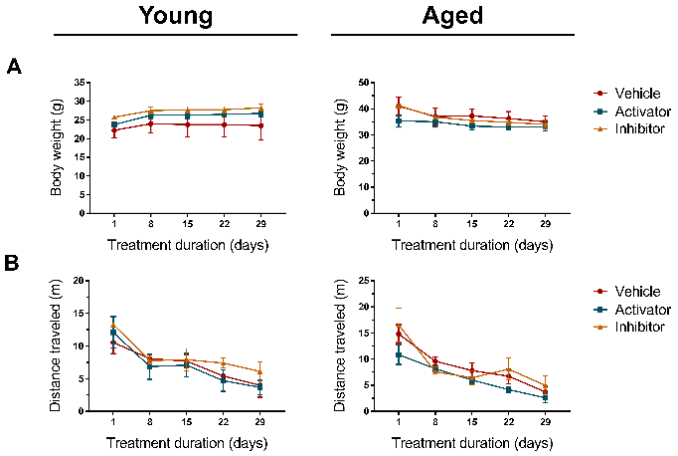
Long-term administration of AMPK inhibitor and activator does not alter the mouse’s vital signs. Young and aged mice were subjected to intraperitoneal administration of vehicle (0.9% saline), inhibitor (Compound C, 10 mg/kg/day), or activator (AICAR, 500 mg/kg/day) for 28 days. Measurements for (A) body weight and (B) spontaneous locomotor activity were taken every week at 9 am, n = 4 per group, Two-way ANOVA (between-groups factors: treatment, treatment duration) was performed but we found no significant interactions.

Long term administration of an AMPK-altering drug such as Compound C and AICAR have the potential to affect an animal's body weight and behavior. Therefore, we monitored the body weight and spontaneous locomotor activity (LMA) of inhibitor- and activator-treated young and aged mice for a period of 28 days. Measurements were taken at baseline (Day 1) and every 7 days following that till the day of sacrifice (Day 29). No significant interactions between Treatment and Treatment duration for body weight (Figure 8A) and LMA (Figure 8B) were observed, which corroborates with another report that administered metformin (AMPK activator) in aged male mice and saw no changes to body weight [66]. It is worth mentioning that the decrease in distance traveled for both young and aged mice can be attributed to the loss of novelty and the mice being increasingly comfortable in that setting/environment.

## CONCLUSIONS

Understanding the fundamental processes that regulate the age-related decline of neurogenesis has long been a goal in the stem cell and aging fields. The idea of increasing the number of endogenous stem cells as a potential therapy for the aging brain is particularly enticing when a similar outcome could be accomplished without the need for invasive procedures such as cell transplantation. Our results revealed that the activation of AMPK signaling was pivotal in the age-related decline of hippocampal neurogenesis, which was substantiated with our finding that i) AMPK isoforms were expressed in the two neurogenic regions, subgranular zone (SGZ) and subventricular zone (SVZ); ii) AMPK signaling was activated in all neural stem cell types and were increased in the aged SGZ and SVZ; (iii) Short term administration of Compound C inhibited AMPK signaling to increase neurogenesis in the aged hippocampus. Taken together, these results indicated that the increase in AMPK signaling with age is a key factor leading to the age-related decline of hippocampal neurogenesis and thus, increased susceptibility to age-associated neurological diseases.
